# Heat treatment of emicizumab samples – a way forward?

**DOI:** 10.1016/j.rpth.2026.106872

**Published:** 2026-07-18

**Authors:** Adrian Kimiaei, Annica Braf, Jovan Antovic

**Affiliations:** 1 Department of Clinical Chemistry, Karolinska University Hospital, Stockholm, Sweden; 2 Department of Molecular Medicine and Surgery, Karolinska Institute, Stockholm, Sweden

As a laboratory measuring emicizumab concentration using a chromogenic substrate assay (CSA) with human reagents (hCSA), interference from residual or exogenous factor (F)VIII activity is an important issue. A recent publication by Müller et al. [1], together with an earlier study by the same group, Hamedani et al. [2], provide an important evaluation of different approaches to mitigating this interference. The results obtained with different types of FVIII-neutralizing inhibitors, either monoclonal antibodies or native FVIII inhibitor plasma, are highly promising, although the method requires additional laboratory steps using specialized reagents and thus may significantly increase operational complexity and costs.

Our laboratory has previously used heat treatment to inactivate FVIII, an approach that Hamedani et al. [2] found to perform well in spiked samples using lyophilized FVIII-deficient plasma. However, they observed relative errors between −59.2% and −30.4% in patient samples, indicating a substantial loss of emicizumab activity. Additional analyses in 2 persons with severe hemophilia A receiving only emicizumab, as well as spiking experiments using nonlyophilized FVIII-deficient plasma and plasma derived from persons with severe hemophilia A and no current treatment, showed similar results, with clinically unacceptable loss of emicizumab activity.

In contrast, our group has previously shown that heat treatment for 30 minutes at 56 °C may be a promising approach for isolating emicizumab activity in the presence of FVIII [3]. In that study [3], residual FVIII after the heat treatment protocol was below 2 IU/dL in all cases, and the sum of FVIII activity and emicizumab-based estimated FVIII activity aligned well with the total FVIII activity before heat treatment. Samples treated for 60 minutes showed a lower measured total activity, suggesting loss of emicizumab activity. However, as emicizumab-based estimated FVIII is likely not linearly convertible to FVIII [4], these findings should be interpreted as preliminary. In light of the inconsistent results reported, the discrepancies in our own observations, and the potential advantages of this simpler approach, we undertook a reevaluation of heat treatment.

As all heat treatments seem to have some effect on emicizumab activity, we aimed to find the shortest incubation time at 56 °C that would allow sufficient FVIII removal. Incubation times of 20 and 25 minutes were evaluated. Leftover anonymized blood samples from routine clinical testing were collected. They had been sampled in 3.2% sodium citrate tubes, centrifuged within 1 hour at 3000 × *g* for 10 minutes at 15 °C, and stored at −70 °C until analysis. To determine the shortest time needed to remove FVIII activity, 10 samples were included: 5 from patients with efmoroctocog alfa treatment (endogenous FVIII activity assessed during a treatment-free period ≤1 IU/dL) and 5 with only endogenous FVIII activity. To evaluate emicizumab stability during heat treatment, 12 samples from emicizumab-treated patients without detectable FVIII activity (defined as a bovine CSA [bCSA] ≤ 1 IU/dL) were analyzed before and after a 30-minute heat treatment at 56 °C. Following initial analyses, a second independent cohort of 12 emicizumab-treated samples without detectable FVIII activity was analyzed using the same protocol. bCSA was measured using Chromogenix reagents (Werfen), and hCSA was measured using BIOPHEN FVIII:C reagents (Hyphen BioMed) and the Emicizumab Calibrator (R2 Diagnostics), both on a Sysmex CS-2500 coagulation analyzer. Only single measurements were performed due to limited sample material. This study was performed in accordance with the Declaration of Helsinki. As only fully anonymized samples were used, ethical approval from our local ethics review authority was not needed.

The heat treatment results are presented in Tables 1 and 2. For removal of FVIII activity, including efmoroctocog alfa, 20 and 25 minutes of heating were insufficient, with several samples showing residual activity > 2 IU/dL, reaching up to 17.6 IU/dL. Following 30 minutes of heat treatment, emicizumab concentrations decreased compared with baseline, with a median absolute relative error (|RE|) of 11% (range, 0%-49.9%) in the initial cohort. Four samples showed marked deviations from baseline, with |RE| between 30.2% and 49.9%. Internal quality control measurements were within predefined limits, and no analytical or preanalytical cause could be identified. Owing to the limited remaining material, repeat testing of these samples was not possible, and because of the anonymized nature of the samples, patient-specific factors could not be investigated.

**Table 1:** Emicizumab concentration before and after heat treatment at 56 °C for 30 minutes.

Bovine CSA FVIII (IU/dL)	Emicizumab preheat (mg/L)	Emicizumab postheat (mg/L)	|RE| (%)
0.1	42.93	39.45	8.8
0.3	38.03	39.75	4.3
0.4	40.88	40.35	1.3
0.2	51.99	37.88	37.2
0.3	36.54	24.38	49.9
1.0	18.08	12.69	42.5
0.1	37.95	33.04	14.9
0.1	33.75	29.84	13.1
0.6	10.70	10.32	3.7
0.0	22.33	20.68	8.0
0.0	45.37	45.37	0.0
0.0	9.76	13.99	30.2
0.1	10.30	7.53	36.8
0.2	8.17	8.70	6.1
0.2	22.26	24.58	9.4
0.2	10.37	9.16	13.2
0.1	9.63	9.16	5.1
0.3	22.72	20.29	12.0
0.3	25.67	25.83	0.6
0.1	12.36	12.02	2.8
0.2	17.60	16.43	7.1
0.2	11.81	10.64	11.0
0.2	12.64	11.12	13.7
0.2	19.35	18.57	4.2

All FVIII measurements were performed by CSA with bovine reagents, and emicizumab measurements were performed by CSA with human reagents and an emicizumab-specific calibrator (results shown in mg/L).

CSA, chromogenic substrate assay; FVIII, factor VIII; |RE|, absolute relative error.

**Table 2:** Factor VIII measurements after 20 and 25 minutes of heat treatment at 56 °C.

FVIII (IU/dL)	20 min	25 min	Efmoroctocog alfa
113.6	0.026	0.014	Yes
74.2	0.019	0.024	Yes
43.5	0.018	0.035	Yes
199.7	0.075	0.115	Yes
15.1	0.003	0.009	Yes
215.0	0.128	0.170	No
166.6	0.011	0.014	No
272.5	0.176	0.159	No
177.9	0.019	0.015	No
217.0	0.042	0.051	No

All measurements were performed with a chromogenic substrate assay with bovine reagents. Results are displayed in IU/dL.

FVIII, factor VIII.

In the independent reevaluation cohort, 12 emicizumab-treated samples were analyzed using the same protocol. In this cohort, the median |RE| was 8.3% (range, 0.6%-36.8%), with only 1 sample showing a deviation exceeding 30%. These findings suggest that the marked deviations observed in the initial cohort may not be representative of the method’s typical performance, as the overall distribution of relative errors was considerably narrower in the independent cohort, although occasional larger discrepancies cannot be excluded.

We observed a modest negative bias in emicizumab concentrations following heat treatment, which appears to be of limited clinical relevance and contrasts with the findings reported by Hamedani et al. [2]. We consider several potential explanations for this discrepancy, the most likely being the incubation time. No specific rationale was provided for the use of 40 minutes, whereas shorter incubation times of 30 minutes have been shown to inactivate FVIII while preserving immunoglobulin G4 antibodies, such as emicizumab, and have been recommended for related applications such as the Nijmegen–Bethesda assay [[5], [6], [7]].

Furthermore, there are several important considerations. Our emicizumab cohort all had bCSA FVIII < 1 IU/dL, which introduces matrix heterogeneity compared with samples containing both emicizumab and FVIII from other sources. However, this seems unlikely to explain the discrepancy, as Hamedani et al. [2] also showed poor emicizumab recovery in a few samples with no FVIII activity. Differences in assay methodology are similarly unlikely to account for the findings; although differential heat sensitivity between modified one stage assay and hCSA could be hypothesized due to matrix effects, comparable results were obtained using liquid chromatography tandem mass spectrometry, which should not be affected. The reduced recovery reported by Hamedani et al. [2] was consistent across samples spiked with exogenous and endogenous FVIII, arguing against a single source of FVIII as the primary explanation. Finally, although there does not seem to be an obvious difference in preanalytical conditions, including sample handling, freezing, thawing, and heating, this cannot be discounted.

Several limitations preclude definitive conclusions regarding heat treatment in our experiments, including the small sample size, large |RE| heterogeneity, and the lack of access to liquid chromatography tandem mass spectrometry, which limited evaluation of samples containing both emicizumab and FVIII. However, as the use of FVIII mimetic antibodies is likely to increase, and given the complexity and limited availability of inhibitor-based FVIII neutralization, we believe our preliminary findings with 30-minute heat treatment are promising and warrant further systematic evaluation of heat treatment protocols and preanalytical factors for removing FVIII interference in FVIII mimetic antibody measurements.
